# Delay of left ventricular longitudinal expansion with diastolic dysfunction: impact on load dependence of e′ and longitudinal strain rate

**DOI:** 10.14814/phy2.12082

**Published:** 2014-07-17

**Authors:** Hiroyuki Iwano, Min Pu, Bharathi Upadhya, Brett Meyers, Pavlos Vlachos, William C. Little

**Affiliations:** 1 Division of Cardiology, University of Mississippi Medical Center, Jackson, Mississippi, USA; 2 Cardiology Section, Wake Forest School of Medicine, Winston‐Salem, North California, USA; 3 Department of Mechanical Engineering, Virginia Tech, Blacksburg, Virginia, USA; 4 School of Mechanical Engineering, Purdue University, West Lafayette, Indiana, USA

**Keywords:** Echocardiography, heart failure, intra left ventricular pressure difference, left ventricular diastolic function

## Abstract

The effect of diastolic dysfunction (DD) on the timing of left ventricular (LV) diastolic longitudinal and circumferential expansion and their load dependence is not known. This study evaluated the timing of the peak early diastolic LV inflow velocity (E), mitral annular velocity (e′), and longitudinal and circumferential global strain rates (SR_E_) in 161 patients in sinus rhythm. The intraventricular pressure difference (IVPD) from the left atrium to the LV apex was obtained using color M‐mode Doppler data to integrate the Euler equation. The diastolic function was graded according to the guidelines. In normals (*N* = 57), E, e′, longitudinal SR_E_, and circumferential SR_E_ occurred nearly simultaneously during the IVPD. With DD (*N* = 104), e′ and longitudinal SR_E_ were delayed occurring after the IVPD (e′: 18 ± 23 msec, longitudinal SR_E_: 13 ± 21 msec from the IVPD), whereas circumferential SR_E_ (−8 ± 28 msec) and E (−2 ± 13 msec) were not delayed. The normal dependence of e′ and longitudinal SR_E_ on IVPD was reduced in DD; while the relation of circumferential SR_E_ and E to IVPD were unchanged in DD. Thus, normally, the LV expands symmetrically during early diastole and both longitudinal and circumferential expansions are related to the IVPD. With DD, early diastolic longitudinal LV expansion is delayed, occurring after the IVPD and LV filling, resulting in their relative independence from the IVPD. In contrast, with DD, circumferential SR_E_ and mitral inflow are not delayed and their normal relation to the IVPD is unchanged.

## Abstract (precis)

In patients with diastolic dysfunction, longitudinal LV expansion is delayed occurring after mitral flow and the termination of the pressure difference from the left atrium to the LV apex. Thus, with diastolic dysfunction longitudinal expansion (e' and longitudinal early diastolic global strain rate) become nearly independent of load.

## Introduction

Early diastolic left ventricular (LV) filling occurs as a consequence of an intraventricular pressure difference (IVPD) from the left atrium (LA) to the LV apex. The peak mitral flow velocity (E) is determined by the IVPD (Cheng et al. 1990). Thus, peak E is reduced with mild diastolic dysfunction in which the IVPD is diminished due to the slowed rate of LV relaxation (Ohno et al. 1994; Little and Oh 2009). With progression of diastolic dysfunction, peak E returns to the normal range (pseudonormalization) or above normal due to an increased IVPD from LA to LV, which is attributed to an increase in LA pressure (Ohno et al. 1994; Little and Oh 2009). Thus, peak E has a biphasic (U shaped) response to diastolic function, reduced with mild diastolic dysfunction, but increased with severe dysfunction associated with elevated LA pressure.

In contrast, the early diastolic mitral annular Doppler velocity (e′) determined by the rate of longitudinal expansion of the LV is progressively reduced with diastolic dysfunction (Little and Oh 2009; Oh et al. 2011). Thus, e′ has been considered a measure of LV relaxation and diastolic function (Nagueh et al. 2001). Normally, e′ occurs coincident with or slightly before E and both peak E and peak e′ are strongly related to the IVPD (Little and Oh 2009). With progressive LV dysfunction, e′ is delayed and occurs after E and the termination of the IVPD. When directly measured in animals, e′ is less dependent on the IVPD in the presence of substantial diastolic dysfunction (Nagueh et al. 2001; Hasegawa et al. 2003). This suggests that normally the LV expands symmetrically with E, and longitudinal and circumferential expansions occurring simultaneously. In contrast, with diastolic dysfunction, the longitudinal expansion may be delayed, occurring after LV filling (E) and circumferential expansion, and after the termination of the IVPD. The recent development of speckle‐tracking echocardiography provides a method to directly test this hypothesis by measuring the myocardial strain rate in longitudinal and circumferential directions (Leitman et al. 2004; Amundsen et al. 2006; Oh et al. 2011). If this hypothesis is correct, it would suggest that the early diastolic longitudinal and circumferential expansions have different dependency on the IVPD from the LA to LV in the presence of diastolic dysfunction. Accordingly, we evaluated (1) the time course of longitudinal and circumferential wall expansions, E wave, and IVPD and (2) dependency of wall expansions on the IVPD in subjects with normal and impaired diastolic function.

## Methods

### Study population

**Figure 1.**
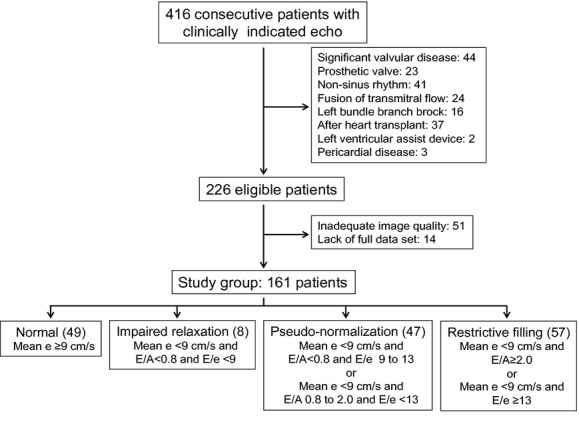
Flowchart of the patient selection and the grading of the diastolic function.

We analyzed consecutive patients who underwent clinically indicated transthoracic echocardiography using the same ultrasound system (Vivid E9, GE Vingmed, Horten, Norway) at Wake Forest Baptist Medical Center from January 2012 to May 2013. Patients with significant left‐side valvular disease, prosthetic valve, pericardial disease, LV assist device, nonsinus rhythm, left bundle branch block, fusion of early and late diastolic mitral inflow, and patients after heart transplantation were excluded. From 226 patients who were eligible for the study inclusion, 51 patients with inadequate echocardiographic image quality and 14 patients who lacked full data sets were also excluded. Accordingly, this study consisted of 161 patients (Fig. 1). The study protocol was approved by the Institutional Review Board of Wake Forest School of Medicine (IRB00012599).

### Two‐dimensional and Doppler echocardiography

Echocardiography was performed by using Vivid E9 ultrasound system with 1.5–4 MHz phased‐array transducer. Digital two‐dimensional cine loops were obtained in the apical 4‐chamber, 2‐chamber, and long‐axis views and mid‐ventricular short‐axis view. The frame rates of cine loops for speckle‐tracking analysis were 61 ± 10 (range 50–91) sec^−1^.

**Figure 2.**
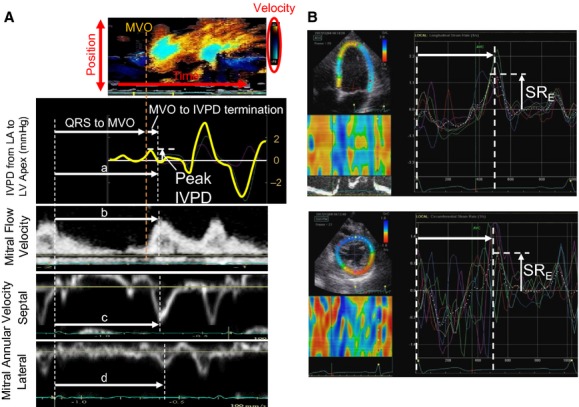
(A) The intraventricular pressure difference (IVPD) from the LA to the LV apex (second top panel) was extracted from the color M‐mode Doppler image (top panel) by integrating the Euler equation. The white vertical arrow indicates the early diastolic peak of the IVPD. The time from the peak QRS wave to the termination of IVPD (a), to the peak E wave (b), and peak septal (c) and lateral e′ (d) were measured. (B) The longitudinal strain rate (SR) (above) and circumferential SR (below) curves are displayed. The colored lines indicate segmental SRs and the white dashed curves indicate global SR. The early diastolic peak of global SR (SR_E_) and time from QRS to SR_E_ (white horizontal arrows) were measured. LA, left atrium; LV, left ventricular; E, early diastolic mitral flow; e′, early diastolic mitral annular velocity; MVO, mitral valve opening.

LV end‐diastolic volume, end‐systolic volume, and ejection fraction were measured from the apical 4‐ and 2‐chamber images using the biplane method of disks. LV mass was calculated according to the Devereux formula (Devereux et al. 1986). The Doppler LV outflow was recorded in the apical long‐axis view, and the time from the peak of the QRS wave to the aortic valve closure (AVC) was measured. Transmitral Doppler flow was recorded in the apical 4‐chamber view, and E, the peak atrial velocity (A), and E/A ratio were measured. Septal and lateral e′ as well as the peak systolic annular velocities (s′) were measured from the apical 4‐chamber view by using the pulsed‐wave tissue Doppler imaging and the average of septal and lateral velocities were used for subsequent analysis. The ratio of E to e′ (E/e′) was calculated. The time from the peak of the QRS wave to the onset of E wave, that to e′ onset, and their difference (T_E‐e′_) were measured as previously reported (Rivas‐Gotz et al. 2003). The time from the QRS wave to peak E, septal e′, and lateral e′ were also measured (Fig. 2). Color M‐mode Doppler (CMMD) images were recorded with a cursor parallel to the LV inflow in the apical 4‐chamber view.

### Definition of the diastolic function

Diastolic function was graded as normal (NL), impaired relaxation (IR), pseudonormal (PN), and restrictive filling (RF) using e′, E/A, and E/e′ according to the European Association of Echocardiography/American Society of Echocardiography recommendations (Fig. 1; Nagueh et al. 2009). In addition, they were further divided into two groups (“normals” and “diastolic dysfunction”) according to the presence or absence of the delay of longitudinal wall expansion to compare the load‐dependency of wall expansion.

### Analysis of the LA to LV pressure difference

The IVPD from the LA to the LV apex was measured using the CMMD data to integrate the Euler equation as previously described (Stewart et al. 2011). The pressure difference at each point along with the scan line was measured relative to the position of the mitral annulus just before mitral valve opening by calculating the line integral between them (Greenberg et al. 2001; Thomas and Popovic 2005; Yotti et al. 2005). The temporal profile of the IVPD from the LA to the LV apex was calculated from the relative pressures similar to the calculations of Greenberg et al. (2001)) and Rovner et al. (2003). From the temporal IVPD profile, we measured the early diastolic peak of the IVPD as well as time from mitral valve opening to the termination of the IVPD (Fig. 2). Then, the time from the peak of the QRS wave to the termination of IVPD was calculated as the time from QRS to mitral valve opening plus the time difference from mitral valve opening to the IVPD termination.

### Speckle‐tracking analysis

The myocardial strain rate (SR) was analyzed offline using an EchoPac workstation (GE Vingmed). The endocardial border was manually traced and then the thickness of the myocardial region of interest was adjusted to include the entire myocardium. The software then tracked the myocardial motion on subsequent frames and the time‐global SR curves, which were calculated with the use of the entire LV wall in the image, were extracted. The time‐longitudinal SR curve was obtained from the apical 4‐chamber, 2‐chamber, and long‐axis views, and the time‐circumferential SR curve was from the midventricular short‐axis view. The peak early diastolic global SR (SR_E_) and the time from the peak of QRS wave to SR_E_ were measured for longitudinal and circumferential parameters (Fig. 2). The timings of the all events were normalized to AVC and expressed as the percentage of the duration of systole (%systole; Cheng‐Baron et al. 2010) as well as the absolute values. The peak systolic global SR was also measured on the global longitudinal SR curves. All parameters were measured from two consecutive cardiac cycles and averaged. Longitudinal parameters from the three apical views were also averaged and used for the final analysis.

### Reproducibility analysis

The test to re‐test reproducibility of the time measurement was assessed in 20 of the subjects. The time from the peak QRS wave to peak E and that to peak e′ were measured from the same Doppler images on two separate days and compared. Similarly, global SR curves were extracted and the time from the QRS to peak longitudinal SR_E_ and to the circumferential SR_E_ were determined on two separate days. The mean and SD of the absolute difference between repeated measurements was 6 ± 6 msec for peak E, 5 ± 6 msec for peak septal e′, 5 ± 6 msec for lateral e′, 4 ± 4 msec for longitudinal SR_E_, and 6 ± 8 msec for circumferential SR_E_. As a result, the absolute difference of repeated measures of the time from peak E to the peak septal e′, peak E to lateral e′, and time taken from circumferential SR_E_ to longitudinal SR_E_ was 7 ± 6 msec, 7 ± 6 msec, and 7 ± 8 msec, respectively.

### Statistical analysis

Continuous variables were expressed as mean ± SD and compared with the 2‐tailed Student *t* test for paired and unpaired data. Demographic continuous parameters and echocardiographic parameters were compared among the different diastolic grades by using one‐way analysis of variance (ANOVA), and post hoc analysis was then performed by using Dunnett's test. Linear regression analysis was carried out for the detection of correlation between the parameters of LV wall expansion or peak E and IVPD. The slopes of the regression lines were compared by analysis of covariance (ANCOVA). For all tests, a *P* value of <0.05 was considered significant. All data were analyzed using JMP software (SAS Institute Inc., Cary, NC).

## Results

### Patient characteristics

Among the 161 patients, diastolic function was classified as NL in 49 patients, as IR in eight patients, as PN in 47 patients, and as RF in 57 patients. The characteristics of the patients are summarized in Table 1. Patients in PN and RF were older than those in NL. Most of the patients with NL had no symptoms of HF, whereas more than half of the patients in PN and RF had HF symptoms. LV mass was greater in PN and RF than in NL. LV ejection fraction was lower in IR, PN, and RF than in NL. The LA diameter was significantly larger in PN and RF than in NL. As expected, e′ was lower in IR, PN, and RF than in NL and E/e′ was higher in PN and RF than in NL. T_E‐e′_ was significantly longer in PN and RF than in NL but it was not prolonged in IR.

**Table 1.** Clinical and echocardiographic characteristics of the study subjects

	Normal	IR	PN	RF	*P* value
*n*	49	8	47	57	
Age, years	48 ± 19	52 ± 13	58 ± 16^1^	59 ± 14^2^	0.003
Male	31 (63)	7 (88)	28 (60)	31 (54)	0.52
Body surface area, m^2^	1.88 ± 0.23	1.99 ± 0.25	1.98 ± 0.25	1.95 ± 0.27	0.34
Systolic blood pressure, mmHg	128 ± 26	135 ± 35	133 ± 20	135 ± 23	0.47
Diastolic blood pressure, mmHg	73 ± 12	78 ± 20	70 ± 10	74 ± 14	0.46
Heart rate, bpm	73 ± 16	75 ± 8	65 ± 9^2^	74 ± 12	0.002
NYHA functional class					
I	43 (88)	2 (25)	18 (38)	14 (25)	<0.0001
II	6 (12)	6 (75)	28 (60)	32 (56)	
III or IV	0 (0)	0 (0)	1 (2)	11 (19)	
Cardiac disease, *n* (%)					
Ischemic heart disease	4 (8)	1 (13)	11 (23)	12 (21)	0.0001
Nonischemic dilated cardiomyopathy	9 (18)	2 (25)	17 (36)	24 (42)	
Hypertensive heart disease	9 (18)	4 (50)	13 (28)	14 (25)	
None	27 (55)	1 (13)	5 (11)	6 (11)	
Others	0 (0)	0 (0)	1 (2)	1 (2)	
Comorbidity, *n* (%)					
Hypertension	20 (41)	5 (63)	20 (43)	38 (67)	0.07
Diabetes Mellitus	12 (24)	0 (0)	7 (15)	19 (33)	0.03
Dyslipidemia	16 (33)	2 (25)	11 (23)	13 (23)	0.67
Echocardiography					
Two‐dimensional findings					
Left atrial diameter, mm	36 ± 7	38 ± 7	40 ± 6^1^	45 ± 9^2^	<0.0001
LV mass index, g/m^2^	85 ± 21	106 ± 26	108 ± 35^2^	124 ± 38^2^	<0.0001
LV end‐diastolic volume, mL	100 ± 27	133 ± 45	132 ± 60^1^	158 ± 72^2^	<0.0001
LV end‐systolic volume, mL	41 ± 17	78 ± 30	73 ± 48^2^	101 ± 66^2^	<0.0001
LV ejection fraction	0.59 ± 0.09	0.42 ± 0.09^2^	0.48 ± 0.14^2^	0.41 ± 0.16^2^	<0.0001
Doppler findings					
E wave velocity, m/s	0.83 ± 0.18	0.47 ± 0.09^2^	0.62 ± 0.11^2^	0.88 ± 0.24	<0.0001
A wave velocity, m/s	0.58 ± 0.16	0.72 ± 0.17	0.71 ± 0.15^2^	0.65 ± 0.27	0.017
E/A	1.54 ± 0.55	0.67 ± 0.08^2^	0.92 ± 0.26^2^	1.72 ± 1.06	<0.0001
E wave deceleration time, msec	196 ± 46	236 ± 42	235 ± 44^2^	189 ± 60	<0.0001
s′, cm/s	8.0 ± 1.8	6.3 ± 1.7^2^	5.9 ± 1.4^2^	4.8 ± 1.5^2^	<0.0001
e′, cm/s	11.8 ± 2.2	6.7 ± 1.3^2^	6.2 ± 1.3^2^	5.2 ± 1.6*	<0.0001
E/e′	7.2 ± 1.9	7.2 ± 1.4	10.2 ± 1.6^2^	17.7 ± 4.6^2^	<0.0001
T_E‐e′_, msec	−2 ± 19	2 ± 17	19 ± 24^2^	26 ± 28^2^	<0.0001

*P* values are for the analysis of variance (ANOVA). IR, impaired relaxation; PN, pseudonormal; RF, restrictive filling; NYHA, New York Heart Association; LV, Left ventricular, E, early diastolic peak of mitral inflow; A, atrial peak of mitral inflow; s′, peak systolic mitral annular velocity; e′, early diastolic mitral annular velocity; E/e′, ratio of E to e′; T_E‐e′_,time from the onset of E wave to e′ onset.

^1^ *P *< 0.05 versus normal group.

^2^ *P *< 0.01 versus normal group by Dunnett's post hoc test.

### Comparison of the time‐measurements

**Figure 3.**
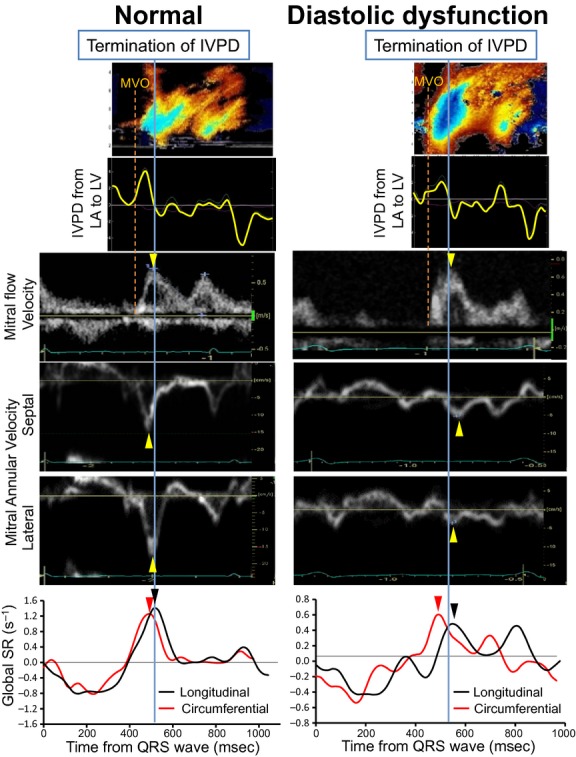
Color M‐mode Doppler image, IVPD from LA to LV, mitral flow velocity, global longitudinal SR curve, and global circumferential SR obtained from a patient with normal diastolic function (Normal) (left) and a patient with restrictive filling (Diastolic Dysfunction) (right). In the normal, the peak e′ and longitudinal and circumferential SR_E_ occur coincident with or prior to the peak mitral flow velocity (E) and the termination of the IVPD. In contrast, in diastolic dysfunction, e′ and longitudinal SR_E_ were delayed occurring after E and after the termination of the IVPD whereas E and the circumferential SR_E_ were not delayed. Abbreviations are the same as Figure 2.

**Figure 4.**
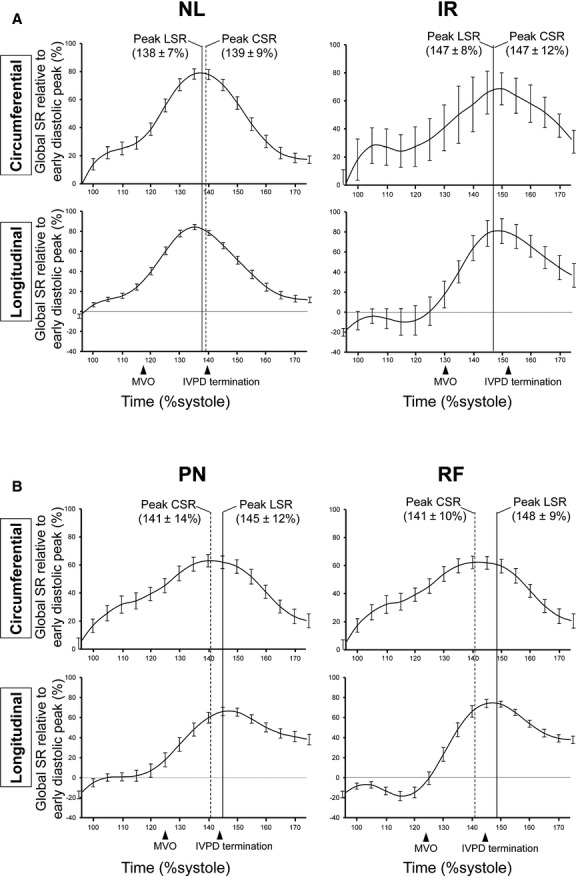
Group‐averaged global SR curves with standard errors are presented. Because the absolute values of SR are varied among the patients, the global SR was expressed as a percentage relative to the patient's early diastolic peak of global SR. This relative global SR was averaged at every 5% of the %systole in each diastolic grade. LSR, global longitudinal strain rate; CSR, global circumferential strain rate. Other abbreviations are the same as Figures 2, 4.

**Figure 5.**
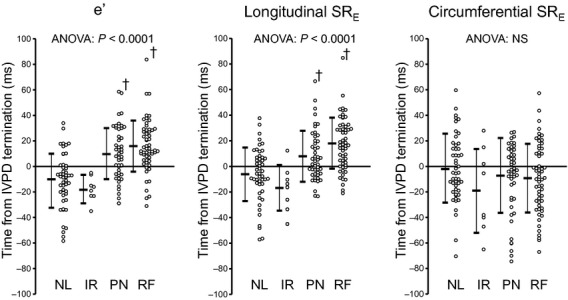
Comparisons of time from the termination of the IVPD to e′, longitudinal SR_E_, and circumferential SR_E_. NL, normal; IR, impaired relaxation; PN, pseudonormal; RF, restrictive filling. Other abbreviations are the same as Figure 2. ^†^*P *< 0.01 vs. normal.

The results of time‐measurements are summarized in Table 2. As previously reported (Courtois et al. 1988), the termination of the IVPD and peak E occurred simultaneously in all groups. The timing of wall expansion was compared to the termination of the IVPD in each diastolic grade, respectively. In PN and RF, peak e′ and longitudinal SR_E_ occurred significantly later than the termination of the IVPD. In contrast, they occurred coincident with or earlier than IVPD termination in NL and IR (Figs. 3, 4). Thus, the peak of the early diastolic longitudinal wall expansion was delayed and occurred after the LA to LV pressure crossover in the presence of clear diastolic dysfunction. However, circumferential SR_E_ occurred coincident with or slightly earlier than the termination of the IVPD in all groups (*P* = 0.64 in NL, *P* = 0.15 in IR, *P* = 0.08 in PN, *P *< 0.05 in RF). As a result, in patients with clear diastolic dysfunction (PN and RF), e′ and longitudinal SR_E_ occurred later than the IVPD termination (18 ± 23 msec and 13 ± 21 msec, respectively) whereas circumferential SR_E_ preceded the IVPD termination by 8 ± 28 msec (Fig. 5).

**Table 2.** Results of the time‐measurements in different diastolic function groups

	Normal (*n* = 49)	IR (*n* = 8)	PN (*n* = 47)	RF (*n* = 57)
Time from the QRS wave				
Termination of IVPD				
msec	504 ± 48	538 ± 22	550 ± 38	515 ± 58
%systole	139 ± 7	152 ± 8	143 ± 11	144 ± 8
Peak E				
msec	504 ± 45	537 ± 24	551 ± 38	518 ± 59
%systole	139 ± 7	151 ± 9	144 ± 11	144 ± 8
Peak e′^1^				
msec	493 ± 42^2^	521 ± 25^2^	560 ± 40^2^	531 ± 61^2^
%systole	136 ± 6^2^	147 ± 9^2^	146 ± 11^2^	148 ± 9^2^
Peak longitudinal SR_E_				
msec	498 ± 43	521 ± 34^3^	558 ± 41^2^	533 ± 62^2^
%systole	138 ± 7	147 ± 8^3^	145 ± 12^2^	148 ± 9^2^
Peak circumferential SR_E_				
msec	503 ± 49	520 ± 24	543 ± 49	506 ± 67^3^
%systole	139 ± 9	147 ± 12	141 ± 14	141 ± 10^2^

^1^ Average of septal and lateral e′.

^2^ *P *< 0.01 versus termination of IVPD within each diastolic grade. Abbreviations are the same as Table 1.

^3^ *P *< 0.05 versus termination of intraventricular pressure difference (IVPD) from the left atrium (LA) to LV within each diastolic grade.

### Relation of the early diastolic wall expansion and the LV filling velocity to peak the IVPD from the LA to LV

**Figure 6.**
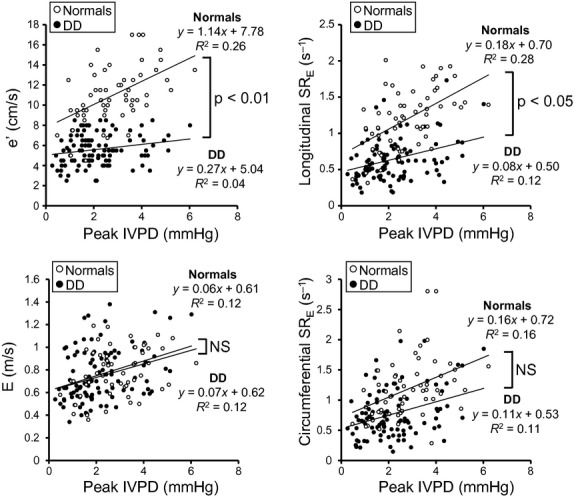
Plots of mean e′, longitudinal SR_E_, peak E wave, and circumferential SR_E_ to peak IVPD from the LA to the LV apex. All correlations were statistically significant. The slope of the relation of e′ and longitudinal SR_E_ versus the IVPD were significantly decreased with diastolic dysfunction (DD) indicating less dependence on the IVPD. In contrast, the slopes of relations of E and circumferential SR_E_ to the IVPD are unaltered with the diastolic dysfunction. Abbreviations are the same as Figure 2.

The correlations of e′, longitudinal SR_E_, circumferential SR_E_, and E to the peak IVPD from the LA to LV were evaluated in NL or IR (“normals”), in which peak e′ and longitudinal SR_E_ occurred before the termination of the IVPD, and in PN or RF (“diastolic dysfunction”), in which they occurred after the IVPD termination, respectively (Fig. 6). The slopes of regression lines of e′ or longitudinal SR_E_ and the peak IVPD were significantly lower in diastolic dysfunction than in normals (e′: 0.27 vs. 1.14 cm/s per mmHg, *P *< 0.01; longitudinal SR_E_: 0.08 vs. 0.18 sec^−1^ per mmHg, *P *< 0.05). In contrast, those of E or circumferential SR_E_ and the peak IVPD were similar between the groups (E: 0.07 vs. 0.06 m/s per mmHg, *P* = 0.85; circumferential SR_E_: 0.11 vs. 0.16 sec^−1^ per mmHg, *P* = 0.33).

### Relation of LV systolic function to early diastolic wall expansion

Consistent with previous results (Yu et al. 2002), s′ and e′ were strongly correlated (*R* = 0.68, *P *< 0.0001). Similarly, the peak systolic global longitudinal SR strongly correlated with longitudinal SR_E_ (*R* = −0.84, *P *< 0.0001).

## Discussion

Our study shows that normally the LV expands symmetrically in early diastole. With diastolic dysfunction, longitudinal expansion delays occurring after LV filling and IVPD. Thus, longitudinal expansion is relatively independent of the IVPD in the presence of diastolic dysfunction.

Since the position of the LV apex remains nearly constant throughout the cardiac cycle (Bowman and Kovacs 2003), the motion of the mitral annulus measured by tissue Doppler imaging reflects LV longitudinal function. Alternatively, this can be assessed as longitudinal strain using speckle tracking. Longitudinal systolic shortening and early diastolic lengthening are reduced in patients with heart failure regardless of ejection fraction (Brucks et al. 2005). In the present study, in normals we found that in early diastole, LV longitudinal and circumferential expansion occur nearly simultaneously with, and are related to the IVPD from the LA to the LV apex as is the transmitral flow (E wave). In contrast, in the presence of diastolic dysfunction, apparent as pseudonormalized or restrictive filling, longitudinal expansion (reflected by e′ and the longitudinal SR_E_) is delayed occurring after circumferential expansion, after the E wave, and after the termination of the IVPD. Thus, in diastolic dysfunction, e′ and the longitudinal SR_E_ are nearly independent of the IVPD, while E and the circumferential SR_E_ remain load dependent. This explains why in the presence of diastolic dysfunction an elevated IVPD due to increased left atrial pressure results in normalization of E and circumferential SR_E_, but not e′ and longitudinal SR_E_. These findings are consistent with the idea that longitudinal LV expansion normally contributes to early diastolic LV filling, while in the presence of diastolic dysfunction longitudinal expansion is a consequence of LV filling since it occurs after the mitral E wave.

During ejection, the annulus is pulled toward the apex which compresses elastic elements in the wall of the LV. Normally, rapid LV relaxation allows the annulus to recoil away from the apex in early diastole (Little 2005). This annular motion and circumferential recoil contribute to the IVPD that progressively extends from the left atrium to the LV apex (Stewart et al. 2011). Thus, the longitudinal expansion is an important contributor to early diastolic filling (Charonko et al. 2013).

With myocardial dysfunction, longitudinal shortening is reduced even in the presence of a normal ejection fraction (Brucks et al. 2005). Several factors may contribute to this loss of longitudinal systolic function. For example, the longitudinally oriented inner myocardial layers are most susceptible to the deleterious effects of interstitial fibrosis (Martinez et al. 2003) and hypoperfusion (Reimer et al. 1977). The resulting reduced longitudinal systolic shortening results in less diastolic recoil. Consistent with this concept, we found a strong correlation between s′ and e′ as well as systolic and early diastolic longitudinal strain rates. Ballo et al. recently reported that, in asymptomatic patients with hypertension, longitudinal systolic function assessed by strain correlated with the diastolic function (e′ and E/e′) better than circumferential systolic function, which is also consistent with this concept. Our results obtained from patients with various systolic functions may extend this concept to the patients with systolic dysfunction. The spherical remodeling in these patients increases the systolic longitudinal relative to circumferential wall stress (Douglas et al. 1989; Rivas‐Gotz et al. 2003), which may cause the delay of longitudinal delay in systolic dysfunction. With severe diastolic dysfunction, the longitudinal expansion occurs after completion of almost all the early diastolic filling (E wave). In this situation, the longitudinal expansion appears to be less load dependent than the circumferential expansion.

Our results are consistent with the observation that the delay of onset of e′ from that of E is an indicator of LV diastolic dysfunction (Rivas‐Gotz et al. 2003; Nagueh et al. 2009). Furthermore, present results suggest the coincidence of circumferential expansion, E wave, and IVPD as well as the load dependency of circumferential SR_E_ in the presence of the diastolic dysfunction. Although circumferential SR_E_ correlates with LV diastolic function (Kim et al. 2011; Meluzin et al. 2011), the load‐dependency of circumferential SR_E_ in both normal and patients with diastolic dysfunction should make it a less useful marker of LV relaxation than longitudinal SR_E_.

## Study Limitations

In this study, we defined diastolic dysfunction as the presence of pseudonormalized or restricted LV filling. The grading of diastolic dysfunction using filling patterns is not always accurate and not all patients can be consistently classified (Unzek et al. 2011). We excluded 24 patients with fused E and A waves, and only included patients in sinus rhythm. Because the sample size of the patients with diastolic grade of IR was only 8 in which seven patients were male and the population contained the limited patients with NYHA class III/IV symptoms, the results are needed to be confirmed in a more balanced population. It is important to recognize that we studied unselected patients whose diastolic dysfunction may have been due to a variety of causes.

We evaluated circumferential SR_E_ from the mid‐LV segments and not apical or basal segments. Furthermore, we evaluated the PD only along a single scan line from the left atrium to the LV apex. Thus, we cannot assess the IVPD and flow outside of the scan line, the interaction of the blood flow with the LV walls, or the impacts of myocardial deformation.

The relatively low sampling rate of speckle‐tracking echocardiography (61 sec^−1^ on average) decreases the ability to measure the timing of SR_E_ in an individual patient and may have contributed to the scatter in our data. In contrast, the pulsed‐wave tissue Doppler determination of e′ has higher temporal resolution making the timing of its measurement more accurate.

## Conclusions

Normally, the LV expands symmetrically during early diastole and both longitudinal and circumferential expansions are related to the IVPD from the LA to the LV apex. Early diastolic longitudinal LV expansion is delayed in the presence of diastolic dysfunction occurring after the termination of the IVPD and early filling. This makes e′ and longitudinal SR_E_ relatively independent of the IVPD in the presence of diastolic dysfunction. In contrast, even in the presence of diastolic dysfunction circumferential wall expansion occurs coincident with and is influenced by the IVPD. Thus, in the presence of diastolic dysfunction, an elevated IVPD due to increased LA pressure results in normalization of E and circumferential SR_E_, but not e′ and longitudinal SR_E_.

## Conflicts of Interest

No conflicts of interest, financial or otherwise, relevant to this study are declared by the authors.
